# Pontine Microtubular Signal Intensity in Hemifacial Spasm: Association with Outcome After Microvascular Decompression Surgery

**DOI:** 10.3390/life16040664

**Published:** 2026-04-14

**Authors:** Hyun Seok Lee, Hong Gee Roh, Won-Jin Moon, Change-Hee Kim, Kwan Park, Jin Woo Choi

**Affiliations:** 1 Department of Neurosurgery, Konkuk University Medical Center, Konkuk University School of Medicine, Seoul 05030, Republic of Korea; 20220205@kuh.ac.kr (H.S.L.); kwanpark@skku.edu (K.P.); 2 Department of Radiology, Konkuk University Medical Center, Konkuk University School of Medicine, Seoul 05030, Republic of Korea; hgroh@kuh.ac.kr (H.G.R.); mdmoonwj@kuh.ac.kr (W.-J.M.); 3 Department of Otorhinolaryngology-Head and Neck Surgery, Konkuk University Medical Center, Research Institute of Medical Science Center, Seoul 05030, Republic of Korea; changhee.kim@kuh.ac.kr; 4 Department of Neurosurgery, Sungkyunkwan University School of Medicine, Seoul 06351, Republic of Korea

**Keywords:** hemifacial spasm, perivascular space, glymphatics, high resolution MRI, high resolution PDI, microvascular decompression

## Abstract

Background: We aimed to investigate the prevalence and clinical significance of pontine microtubular signal intensity (MSI), presumed dilated perivascular or perineural spaces, in patients with hemifacial spasm (HFS) using high-resolution MRI using proton density-weighted imaging (HR-PDI). Methods: We retrospectively analyzed 438 patients with unilateral HFS who underwent microvascular decompression (MVD) and preoperative HR-PDI. MSI was defined as a linear or curvilinear hyperintense lesion along the presumed course of the intraparenchymal facial nerve fascicles within the pons on HR-PDI. The presence and laterality of MSI were evaluated by consensus between two reviewers and classified according to their relationship to the symptomatic side of HFS as ipsilateral (same side as the facial spasm), contralateral (opposite side), or bilateral. Clinical characteristics, surgical findings, and postoperative outcomes were compared according to the presence of ipsilateral MSI. A control group of 307 subjects who underwent HR-PDI for non-central neurologic symptoms was included to assess the prevalence of MSI. Multivariable logistic regression analysis was performed to identify factors associated with immediate postoperative improvement after MVD. Results: MSI was more frequently observed in patients with HFS than in controls after adjusting age and sex (OR, 3.78; 95% CI, 2.747–5.197; *p* < 0.001). Ipsilateral MSI was identified in 267 of 438 patients (61.0%). Patients with ipsilateral MSI showed a significantly higher frequency of contralateral MSI (*p* < 0.001) and vertebralartery-related compression (*p* = 0.002). Immediate postoperative improvement after MVD was less frequent in patients with ipsilateral MSI than in those without MSI (77.5% vs. 86.5%, *p* = 0.019). Multivariable logistic regression analysis demonstrated that ipsilateral MSI was independently associated with a lower likelihood of immediate postoperative improvement (OR, 0.411; 95% CI, 0.222–0.759; *p* = 0.005). However, long-term surgical outcomes were not significantly different according to the presence of MSI. Conclusions: Pontine MSI on HR-PDI is more frequently observed in patients with HFS and is associated with a lower likelihood of immediate postoperative improvement and a tendency toward delayed recovery after MVD but not with poorer long-term outcomes. These findings suggest that MSI may represent microstructural or neurofluidic alterations along the pontine facial nerve pathway and may serve as an imaging marker of delayed recovery dynamics.

## 1. Introduction

Hemifacial spasm (HFS) is a cranial nerve hyperexcitability disorder characterized by involuntary, repetitive, paroxysmal facial contractions that are typically unilateral [1]. Neurovascular compression at the root exit zone (REZ) of the cranial nerve is widely accepted as a critical etiologic factor, and histopathologic studies have described demyelination, vacuolization, and partial axonal degeneration at the REZ, which may facilitate abnormal impulse transmission and ephaptic coupling between adjacent fibers [2]. However, neurovascular compression alone does not fully explain the occurrence of HFS. Brainstem lesions—including tumors, vascular malformations, and inflammatory or infectious processes—can produce HFS [3,4], and neurovascular contact is not infrequently observed on MRI in asymptomatic individuals, whereas some patients with clinically definite HFS lack clear evidence of compression on imaging study [1,5]. These observations suggest that additional predisposing or accompanying factors may influence susceptibility to occurrence of HFS. Accordingly, prior imaging studies have explored macroscopic anatomical contributors such as posterior fossa volume and crowdedness, the distance between the brainstem and internal acoustic meatus, and the angulation between the facial nerve and brainstem using conventional MRI [6,7,8,9,10,11]. While these factors support the concept that the spatial relationships among the facial nerve, brainstem, and posterior fossa likely play a role in HFS, there is very little research on the microstructural changes in the pontine fascicular segment of the facial nerve and adjacent pontine parenchyma [12]. High-resolution MRI (HR-MRI), referring to high-spatial-resolution imaging technique, including heavily T2-weighted sequence or proton density-weighted image (HR-PDI), is essential for evaluating the complex neurovascular relationship in HFS [4]. However, while previous studies have focused on the morphological changes in the REZ and cisternal segments, parenchymal abnormalities—which are only visible on HR-MRI—have been largely overlooked and undervalued [6,7,8,10]. In clinical practice, we frequently observe hyperintense foci (dot-like or curvilinear) along the lateral pons in HFS patients—findings not evident on routine MRI. These lesions are presumed to represent dilated pontine perivascular and/or perineural spaces. As integral parts of the glymphatic system, alterations in these spaces may reflect impaired clearance and tissue vulnerability that may cause multiple neurologic conditions [13,14,15]. Recent evidence in trigeminal neuralgia suggests that reduced glymphatic function contributes to nerve hyperexcitability [16,17]. According to these considerations, we hypothesized that alterations in interstitial or glymphatic fluid dynamics may arise from multiple contributing factors, including neurovascular compression, and may be reflected as dot-like, linear, or curvilinear hyperintensities in the pons on HR-PDI, presumed to represent dilated perivascular and/or perineural spaces. Therefore, this study investigated the relationship between these presumed dilated pontine perivascular/perineural spaces and HFS, assessing their clinical significance of their presence to predict surgical outcome after microvascular decompression (MVD).

## 2. Materials and Methods

This study was approved by the Institutional Review Board of our hospital, and the requirement for informed consent was waived (KUMC 2023-05-069). Data were retrospectively collected from the electronic medical records of consecutive patients with HFS who underwent MVD performed by a single neurosurgeon (K.P.) between January 2021 and September 2023. A total of 443 consecutive patients with HFS who underwent MVD during the study period were initially identified. Among these, 5 patients were excluded because preoperative HR-PDI was not available due to MRI being performed at outside institutions. Accordingly, 438 patients were included in the final imaging analysis.

### 2.1. Clinical and Surgical Data

Demographic and clinical data such as age at the time of MVD, sex, disease duration, involved side, degree of HFS, and comorbidities were collected. The surgical findings such as offending vessels and compression pattern were reviewed as reported previously. The presence of discoloration and degree of indentation to the facial nerve REZ by compressing vessels were assessed in the surgical field by one neurosurgeon who had experience of over 20 years in MVD. The degree of indentation was categorized into four grades: grade 1, close contact without indentation; grade 2, mild indentation; grade 3, moderate indentation; and grade 4, severe indentation with cranial displacement of the 7th cranial nerve over the 8th. Surgical outcomes and complications were usually assessed postoperatively. Patients’ outcomes were classified into immediate, delayed improvement, persistent and recurrent spasm. Improvement was defined as the complete disappearance of spasm or minimal residual spasm (less than 10% of spasm compared with the presurgical condition) based on the subjective evaluation of each patient. Immediate response was defined as the improvement of the spasm within 3 days after the MVD, and delayed improvement over a 4-day to 12-month follow-up period. Recurrence was defined as reappearance of spasm (more than 10% of spasm compared with the presurgical condition) after improvement. Surgical complications including facial palsy and hearing loss were also evaluated according to the previously reported criteria [1,5,18]. Patients were scheduled for follow-up at 1, 3, 6, and 12 months after surgery and annually thereafter. All patients included in the analysis had at least 12 months of follow-up.

### 2.2. MR Imaging: High-Resolution Proton Density-Weighted Images

All patients underwent routine whole-brain MRI, including sagittal T1-weighted imaging (T1WI), axial T2-weighted imaging (T2WI), axial FLuid-attenuated Inversion Recovery (FLAIR), and post-contrast 3D T1WI with axial, coronal, and sagittal reformatted images (slice thickness, 3–4 mm). In addition to these conventional sequences, HR-MRI techniques for cranial nerve evaluation were performed. In this context, HR-MRI refers to imaging approaches with high spatial resolution optimized for assessing cranial nerves and adjacent structures. In the present study, we specifically utilized high-resolution proton density-weighted imaging (HR-PDI), which was focused on the brainstem and the 7th/8th cranial nerves and used for all imaging analyses. Time-of-flight MR angiography (TOF-MRA) of the vertebrobasilar system, including the vertebral, basilar, anterior inferior cerebellar, and posterior inferior cerebellar arteries, was also performed using 3-T MR scanners from two vendors (Magnetom Vida, Siemens Healthineers, Erlangen, Germany; Discovery 750, GE HealthCare, Chicago, IL, USA).

HR-PDI for brainstem and 7th/8th cranial nerves were obtained with a slab thickness of 30–40 mm centered along an imaginary line connecting both internal auditory canals. Three-dimensional FSE sequences with following parameters were used according to each vendor: GE (CUBE; TR/TE, 1502/67.57 milliseconds; flip angle, 90 degrees; echo train length, 54; matrix, 300 × 300 mm; field of view, 180 mm; slice thickness, 0.3 mm; number of averages, 2; acquisition voxel size, 0.6 × 0.6 × 0.6 mm; reconstructed voxel size, 0.35 × 0.35 × 0.3 mm; acquisition time, 6 min 13 s) and Siemens (SPACE, Sampling Perfection with Application-optimized Contrasts by using flip angle Evolution; TR/TE, 2000/30 milliseconds; flip angle, 100 degrees; echo train length, 63; matrix, 320 × 320; field of view, 180 mm; slice thickness, 0.3 mm; number of averages, 2; acquisition voxel size, 0.56 × 0.56 × 0.6 mm; reconstructed voxel size, 0.28 × 0.28 × 0.3 mm; acquisition time, 6 min 34 s).

### 2.3. Image Analysis

**Figure 1:**
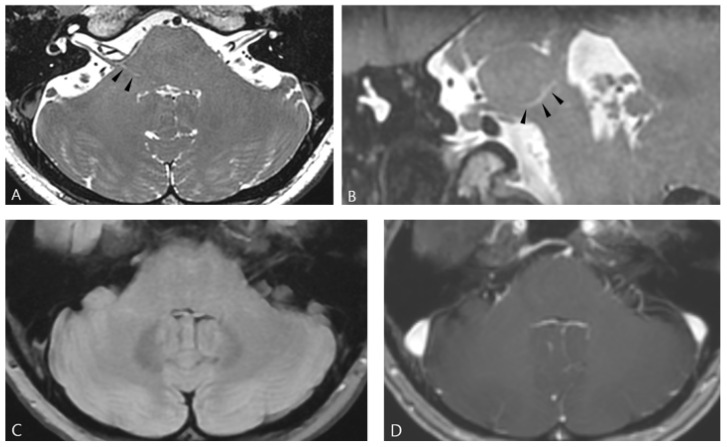
Axial (**A**) and oblique coronal (**B**) high-resolution proton density images (HR-PDI) demonstrate a curvilinear high-signal-intensity lesion along the presumed course of the intraparenchymal facial nerve fascicles in the right lateral pons (arrowheads). Conventional MRI sequences, including axial fluid-attenuated inversion recovery (FLAIR) (**C**) and post-contrast 3D T1-weighted imaging (**D**), show no definite focal pontine lesion.

Routine axial T2WI, FLAIR, and post-contrast 3D T1WI with axial, coronal and sagittal reformatted images were reviewed to exclude the possibility of subacute or chronic subcortical infarction, inflammatory, infectious, demyelinating lesions, or tumors. Image analysis was performed by a neuroradiologist. A visible perivascular or perineural space was defined as a short linear or curvilinear hyperintense lesion observed on HR-PDI along the presumed course of the intraparenchymal facial nerve fascicles (Figure 1). These lesions were not visible on routine MRI sequences that were previously mentioned. In the present study, this imaging finding was termed microtubular signal intensity (MSI), a descriptive imaging term referring to linear or curvilinear hyperintensities observed on HR-PDI, reflecting the tubular appearance of the signal and not implying a specific histopathologic substrate such as microtubular structures or other cellular-level pathology. The presence of curvilinear high-signal-intensity lesions, termed MSI in both the patient and control groups, was determined by consensus between a board-certified neuroradiologist with 16 years of experience and a neurosurgeon with 5 years of experience in microvascular decompression. Both reviewers were blinded to the presence and laterality of HFS and were not involved in MVD in each patient.

### 2.4. Statistical Analysis

To evaluate the significance of MSI in patients with HFS compared with a control group, 307 control subjects (male: female = 152:155; mean age, 66.1 ± 15.8 years) were included. These individuals underwent brain MR including HR-PDI at our institution as part of the evaluation for peripheral symptoms such as hearing disturbance, dizziness, or vertigo. All included subjects had no clinical evidence of central neurologic disorders and showed no apparent structural abnormalities on MRI. Because the number of controls (*n* = 307) was smaller than that of cases (*n* = 438), multivariable logistic regression analysis was performed instead of propensity score matching to preserve statistical power. The model was adjusted for potential confounders, including age and sex.

The patients with HFS were divided into two groups based on the presence of MSI at the ipsilateral side of HFS, and demographic, clinical characteristics, surgical responses, and postoperative complications of the two groups were compared retrospectively. Offending vessels and compression patterns were categorized into four (AICA/PICA, AICA + PICA, VA-related, and venous) and five (one vessel, two vessel, arachnoid/perforator, encircling, and penetrating) groups to reduce sparse data and reflect clinically meaningful compression mechanisms. Compression severity was classified according to the degree of indentation of the facial nerve at the root exit zone. The significance of differences between two groups was evaluated using the independent Student’s *t*-test or the Mann–Whitney U test for continuous and ordinal variables, while Pearson’s chi-square test or Fisher’s exact test was used for categorical variables. To examine whether ipsilateral pontine MSI on HR-PDI is associated with surgical response following MVD, multivariable logistic regression analysis was performed. Variables with potential clinical relevance, including age, sex, duration of symptoms, severity of HFS, presence of ipsilateral and contralateral MSI, offending vessel type, compression degree at the REZ, and REZ discoloration, were entered into a multivariable logistic regression model to identify independent predictors of immediate postoperative improvement. Odds ratios (ORs) with 95% confidence intervals (CIs) were calculated to estimate the strength of association between each variable and surgical outcome. A *p* value < 0.05 was considered statistically significant. All statistical analyses were performed using SPSS software (IBM SPSS Statistics, version 28.0.0).

## 3. Results

### 3.1. Clinical and Surgical Findings

The overall clinical characteristics, surgical findings, complications, and surgical outcomes of the HFS are summarized in Table 1. Patients included in final imaging analysis totaled 438 patients (male: female = 111: 327; mean age; 55.8 ± 11.1 years). The mean duration of symptoms was 61 months (range; 5–360 months, SD; 43.2). Right side was affected in 215 (49.1%) and left in 223 (50.9%) patients. Severity of the HFS was classified as grade I in 3 patients (0.7%), grade II in 165 (37.7%), grade III in 188 (42.9%), and grade IV 82 (18.7%). The most common offending vessel was AICA or PICA, observed in 314 patients (71.7%), followed by vertebral-artery-related vessels in 88 patients (20.1%), combined AICA and PICA compression in 29 patients (6.6%), and venous compression in 7 patients (1.6%). The most common compression pattern was arachnoid or perforator-mediated compression, in which the arachnoid or perforating vessels tether the main offending vessel, observed in 149 patients (34.0%), followed by two-vessel compression in 148 patients (33.8%), single-vessel compression in 119 patients (27.2%), encircling compression in 21 patients (4.8%), and penetrating compression in 1 patient (0.2%). The degree of indentation and discoloration of the REZ could not always be fully evaluated because of the restricted surgical field and the need to minimize injury to adjacent cranial nerves. Among the 438 patients, discoloration was evaluated in 368 patients (84.0%) and indentation in 430 patients (98.2%). Among the 430 patients assessed for indentation, grade I was observed in 17 (4.0%), grade II in 209 (48.6%), grade III in 116 (27.0%), and grade IV in 88 (20.5%). Among the 368 patients evaluated for discoloration, 325 (88.3%) showed REZ discoloration.

Immediate improvement occurred in 355 patients (81.1%), whereas 83 patients (18.9%) showed no immediate response. Among those without immediate improvement, 69 patients (83.1%) experienced delayed improvement during follow-up. Five patients who initially improved experienced recurrence within 12 months. Overall, 419 patients (95.7%) achieved symptom improvement at 1-year follow-up. Postoperative complications included facial palsy in 76 patients (17.4%) and hearing loss in 12 patients (2.7%). Facial palsy occurred immediately in 18 patients and developed in a delayed manner in 58 patients, with gradual improvement during follow-up.

### 3.2. Presence of Pontine Hyperintense Lesion on High-Resolution Proton-Density Imaging

Among the 307 control subjects, MSI was detected in 108 patients (108/307, 35.2%). MSIs were observed on the right side in 60 patients (19.5%), on the left side in 76 patients (24.8%), and bilaterally in 28 patients (9.1%). Among the 438 patients with HFS, pontine MSIs were identified on the ipsilateral side only in 156 patients (35.6%), on the contralateral side only in 25 (5.7%), and bilaterally in 111 (25.3%). No MSI was detected in the remaining 146 patients (33.3%). When stratified by the affected side, among 215 cases of right-sided HFS, ipsilateral (right-only) lesions were observed in 74 patients, contralateral (left-only) lesions in 15, bilateral lesions in 50, and no MSI in 76. Among 223 cases of left-sided HFS, ipsilateral (left-only) lesions were observed in 82 patients, contralateral (right-only) lesions in 10, bilateral lesions in 61, and no MSI in 70. After adjusting for age and sex, patients with HFS showed significantly higher odds of exhibiting any MSI compared with controls (OR, 3.78; 95% CI, 2.747–5.197; *p* < 0.001).

### 3.3. Group Comparison Between Patients with and Without Ipsilateral Pontine MSI

The stratified clinical characteristics, surgical findings, complications, and surgical outcomes according to the presence of MSI are also presented in Table 1. Ipsilateral pontine MSI on HR-PDI was more frequently observed in male patients with HFS (*p* = 0.020). Patients with ipsilateral MSI also demonstrated a significantly higher frequency of contralateral pontine MSI compared with those without ipsilateral MSI (*p* < 0.001). The distribution of offending vessels differed significantly (*p* = 0.002) according to the presence of MSI, and vertebral-artery-related compression was more frequent in patients with MSI (66/267, 24.7%) than in those without MSI (22/171, 12.9%). However, the distribution of compression patterns did not differ significantly according to MSI status (*p* = 0.125). In addition, immediate postoperative improvement was less frequently observed in patients with ipsilateral pontine MSI than in those without MSI (*p* = 0.019).

### 3.4. Relationship Between the Pontine Hyperintense Lesion and Clinical Outcome

Multivariable logistic regression analysis demonstrated that the presence of ipsilateral pontine MSI was independently associated with a lower likelihood of immediate postoperative improvement after MVD (OR, 0.411; 95% CI, 0.222–0.759; *p* = 0.005). Other clinical and surgical variables, including age, sex, duration of symptoms, hemifacial spasm severity, contralateral MSI, offending vessel type, REZ discoloration, and compression degree, were not significantly associated with immediate postoperative improvement (Table 2).

## 4. Discussion

In this study, presumed dilated perivascular or perineural spaces, termed microtubular signal intensity (MSI), were more frequently observed in the ipsilateral pons of patients with HFS than in individuals without HFS. Patients with ipsilateral MSI demonstrated a significantly higher frequency of contralateral MSI. In addition, vertebral-artery-related compression was more frequently observed in patients with ipsilateral MSI, suggesting a possible association between certain vascular configurations and the presence of these lesions. MSI was also more frequently identified in male patients and was associated with a distinct postoperative recovery pattern after MVD, characterized by a lower rate of immediate symptom relief and a tendency toward delayed improvement. However, long-term surgical outcomes were not significantly different according to the presence of MSI.

Previous studies have shown that a greater burden of perivascular spaces is more commonly observed in male individuals, even in the general population [19]. Consistent with this, ipsilateral MSI was more frequently detected in male patients in our cohort. However, male sex itself was not an independent predictor of immediate postoperative improvement in multivariable logistic regression analysis. This suggests that the higher prevalence of MSI in male patients may partly reflect a sex-related predisposition to the development or visibility of perivascular spaces rather than a mechanism specific to HFS alone.

Patients with ipsilateral MSI also demonstrated a significantly higher frequency of contralateral MSI. This finding suggests that the presence of visible perivascular or perineural spaces may not represent a purely focal phenomenon confined to the affected side. Instead, MSI may reflect a more generalized susceptibility or alteration in the neural microenvironment, potentially involving perivascular or perineural spaces, rather than a strictly localized pathological process.

The exact nature of MSI remains uncertain. The perivascular spaces of the brainstem have been less extensively investigated than those in other regions [15,20]. Most MRI studies have described dilated perivascular spaces predominantly in the midbrain, particularly near the cerebral peduncles [21,22,23]. In the upper midbrain, where perivascular spaces are visible at the mesencephalodiencephalic junction, they are typically observed along the posterior (interpeduncular) thalamoperforating artery, the paramedian mesencephalothalamic artery, and the short and long circumferential arteries originating from the upper basilar artery or proximal posterior cerebral artery. Because the pons, like the midbrain, is supplied by numerous small perforating branches arising from circumferential arteries of the vertebrobasilar system, similar perivascular spaces are likely to exist within the pontine parenchyma. However, to our knowledge, detailed anatomical or imaging studies specifically characterizing perivascular spaces in the pons remain limited. Within this anatomical context, MSI may reflect dilatation or alteration of perivascular or perineural spaces related to changes in interstitial fluid dynamics. Perivascular spaces have recently gained attention as important components of the glymphatic system, which facilitates the transport and clearance of metabolic waste products from the brain [13,15,24]. In addition to perivascular pathways, perineural spaces along cranial nerves may also contribute to glymphatic outflow [25]. Importantly, such alterations are unlikely to arise from a single mechanism. Neurovascular compression may be one contributing factor; however, a direct causal relationship cannot be established. In addition, intrinsic susceptibility of the neural microenvironment, as well as other potential influences such as subtle inflammatory changes or structural alterations in the subarachnoid space, may also play a role. Previous studies have reported findings suggestive of low-grade inflammatory or structural changes in patients with HFS, including fibrotic changes in the facial nerve sheath, thickened arachnoid membranes independent of arterial compression, and mildly elevated cytokine levels such as interleukin-6 and interleukin-8 [26,27].

Another possible explanation is that these hyperintense lesions reflect secondary changes related to neural injury. Previous studies have suggested that dilated perivascular spaces may be associated with neural atrophy or demyelination in neurodegenerative disorders such as Alzheimer disease, Parkinson disease, and multiple sclerosis [24,28,29,30]. In one case report describing a dilated perivascular space in the brainstem causing ipsilateral HFS [23], several mechanisms were proposed, including ex vacuo enlargement due to surrounding brain tissue atrophy, coiling of the aging artery in hypertension due to pulsation, altered arterial wall permeability, fibrosis, or obstruction of lymphatic drainage pathways. However, in our cohort, neither older age nor vascular risk factors such as hypertension and diabetes were associated with the presence of MSI, suggesting that MSI may not simply reflect chronic degenerative change. Furthermore, diffusion tensor imaging studies have demonstrated microstructural abnormalities of the affected facial nerve, suggesting that demyelination and axonal injury may occur in HFS [12]. In this regard, differentiation from demyelinating lesions should also be considered. In trigeminal neuralgia, demyelinating plaques—particularly in multiple sclerosis—typically demonstrate additional abnormalities on conventional MRI, including hyperintensity on FLAIR and hypointensity on T1WI, sometimes with contrast enhancement. In contrast, the pontine lesions observed in our cohort were visible only on HR-PDI and were not detectable on routine sequences, including FLAIR and pre- or post-contrast T1WI. This imaging pattern is less typical of classical demyelinating lesions. Nevertheless, subtle intrinsic neural changes such as demyelination cannot be completely excluded in the absence of histologic confirmation. Therefore, these findings suggest that MSI may be interpreted as a non-specific imaging marker of altered neural microenvironment arising from the combined effect of multiple causes.

Although long-term outcomes after MVD for HFS are generally excellent, with reported success rates ranging from approximately 85% to 95% [19,31,32], the clinical course after surgery can be variable. Not all patients become asymptomatic immediately after MVD, and up to 19% of patients continue to experience residual spasms for more than one month postoperatively [1,4]. Previous studies have investigated various factors associated with both short-term and long-term postoperative responses [10,32,33,34,35,36,37]. In the present study, patients with MSI were less likely to show immediate postoperative improvement after MVD but tended to demonstrate delayed recovery, while long-term outcomes were not significantly different between patients with and without MSI. These findings suggest that the presence of pontine MSI may be associated with a slower postoperative recovery pattern rather than a poorer surgical outcome. One possible explanation is that MSI reflects a pre-existing alteration in the neural microenvironment. While MVD effectively addresses the mechanical component of neurovascular compression, normalization of the underlying neural environment may require additional time, which may account for delayed recovery. From a clinical perspective, these findings may have practical implications. The presence of MSI may help explain why some patients do not experience immediate postoperative improvement despite ultimately achieving favorable long-term outcomes. Recognition of this imaging finding may therefore assist in postoperative counseling and support a more conservative observational approach during early follow-up.

Several limitations of this study should be acknowledged. First, the presumption that MSI represents perivascular or perineural spaces was not confirmed histologically. Second, image analysis was performed by consensus rather than independent readings, and interobserver agreement was not formally assessed. Because the identification of MSI required careful evaluation of subtle hyperintense linear structures on HR-PDI, consensus interpretation was adopted to minimize potential misclassification. Third, longitudinal changes in MSI after MVD were not evaluated. Follow-up imaging was not routinely performed due to the retrospective design and clinical practice patterns, and may impose financial burden in asymptomatic patients. Future prospective studies incorporating longitudinal imaging will be important to clarify the biological significance of MSI. In addition, the visibility of perivascular spaces may vary depending on physiological factors such as circadian rhythm, which could complicate the interpretation of longitudinal imaging findings. Finally, differences in baseline characteristics between the HFS and control groups, including age and clinical context, may also have introduced residual confounding despite statistical adjustment.

## 5. Conclusions

Pontine MSI appears to be associated with a lower likelihood of immediate postoperative improvement and a tendency toward delayed recovery after MVD. However, these lesions do not appear to adversely affect long-term surgical outcomes or complication rates. Therefore, MSI may serve as an imaging marker of altered postoperative recovery dynamics rather than a predictor of poor long-term prognosis.

## Figures and Tables

**Table 1:** Comparison of Clinical Characteristics, Surgical Findings, and Outcomes According to Ipsilateral Pontine MSI.

Characteristics	Overall (*n* = 438)	Non-Visible MSI (*n* = 171)	Visible MSI (*n* = 267)	*p*-Value
Age (years)	55.8 ± 11.1	55.1 ± 10.3	56.2 ± 11.6	0.311
Male sex	111 (25.3%)	33 (19.3%)	78 (29.2%)	0.020
Lesion side (Right:Left)	215:223	91:80	124:143	0.166
Hypertension	147 (33.6%)	58 (33.9%)	89 (33.3%)	0.899
Diabetes	46 (10.5%)	18 (10.5%)	28 (10.5%)	0.990
Spasm duration (months)	61.0 ± 43.2	60.7 ± 35.9	61.2 ± 47.4	0.897
Spasm degree (I/II/III/IV)	3/165/188/82	1/61/81/28	2/104/107/54	0.483
Contralateral MSI	136 (31.1%)	25 (14.6%)	111 (41.6%)	<0.001
**Surgical Findings**				
*Offending Vessel*				0.002
AICA or PICA	314 (71.7%)	133 (77.8%)	181 (67.8%)	
AICA and PICA	29 (6.6%)	10 (5.8%)	19 (7.1%)	
VA related	88 (20.1%)	22 (12.9%)	66 (24.7%)	
Venous offender	7 (1.6%)	6 (3.5%)	1 (0.4%)	
*Compression type*				0.125
One vessel type	119 (27.2%)	47 (27.5%)	72 (27.0%)	
Two vessels type	148 (33.8%)	50 (29.2%)	98 (36.7%)	
Arachnoid/Perforator type	149 (34.0%)	61 (35.7%)	88 (33.0%)	
Encircling type	21 (4.8%)	13 (7.6%)	8 (3.0%)	
Penetrating type	1 (0.2%)	0 (0.0%)	1 (0.4%)	
*Compression Degree (I/II/III/IV, n = 430)*	17/209/116/88	6/80/53/29	11/129/63/59	0.299
*REZ discoloration (n = 368)*	325 (88.3%)	131 (92.3%)	194 (85.8%)	0.062
**Surgical Outcomes**				
*Complications*				
Facial palsy	76 (17.4%)	25 (14.6%)	51 (19.1%)	0.227
Hearing loss	12 (2.7%)	4 (2.3%)	8 (3.0%)	0.681
*Post-operative Improvement of spasm*				
Immediate	355 (81.1%)	148 (86.5%)	207 (77.5%)	0.019
Delayed	69 (15.8%)	20 (11.7%)	49 (18.4%)	0.062
Long-term	419 (95.7%)	165 (96.5%)	254 (95.5%)	0.607

MSI, microtubular signal intensity; AICA, anterior inferior cerebellar artery; PICA, posterior inferior cerebellar artery; VA, vertebral artery; REZ, root exit zone.

**Table 2:** Multivariable Logistic Regression Analysis for Factors Associated With Immediate Postoperative Improvement after Microvascular Decompression.

Variable	Odds Ratio	95% Confidence Interval	*p*-Value
Sex (male)	0.933	0.505–1.723	0.825
Age	1.013	0.988–1.039	0.321
Duration of symptoms	0.996	0.990–1.002	0.215
Hemifacial spasm severity grade	1.414	0.960–2.083	0.080
Contralateral MSI	1.616	0.891–2.932	0.114
*Offending Vessel type*			0.860
AICA or PICA versus vein	0.719	0.276–1.873	0.500
AICA + PICA versus vein	0.792	0.405–1.548	0.495
VA related vs. vein	-	-	0.999
Ipsilateral MSI	0.411	0.222–0.759	0.005
REZ discoloration	0.968	0.427–2.194	0.939
Compression degree (indentation)	0.960	0.687–1.341	0.810

MSI, microtubular signal intensity; AICA, anterior inferior cerebellar artery; PICA, posterior inferior cerebellar artery; VA, vertebral artery; REZ, root exit zone.

## Data Availability

The data presented in this study are available on request from the corresponding author.
